# A Rehabilitation Tool Designed for Intensive Web-Based Cognitive Training: Description and Usability Study

**DOI:** 10.2196/resprot.2899

**Published:** 2013-12-13

**Authors:** Vítor Tedim Cruz, Joana Pais, Virgílio Bento, Cátia Mateus, Márcio Colunas, Ivânia Alves, Paula Coutinho, Nelson Pacheco Rocha

**Affiliations:** ^1^Neurology DepartmentHospital São SebastiãoCentro Hospitalar Entre Douro e VougaSanta Maria da FeiraPortugal; ^2^Clinical Research OfficeHealth Sciences DepartmentUniversity of AveiroAveiroPortugal; ^3^Neuropsychology Laboratory, Neurology DepartmentHospital São SebastiãoCentro Hospitalar Entre Douro e VougaSanta Maria da FeiraPortugal; ^4^Maia Institute of Higher EducationMaiaPortugal; ^5^UnIGENeInstituto de Biologia Molecular e CelularUniversity of PortoPortoPortugal

**Keywords:** cognitive training, cognitive deficits, neurorehabilitation, Web-based applications, eHealth systems, usability test

## Abstract

**Background:**

Cognitive deficits are among the most disabling of neurological diseases and have a serious impact on the quality of life of patients and families. Cognitive training has been proven successful in improving or compensating for neuropsychological deficits after acute brain injury, but its efficacy highly depends on the intensity of treatment over an extended period of time. Therefore, cognitive training indicates expensive human resources and renders the rehabilitation process vulnerable to physical and economic barriers for the majority of patients.

**Objective:**

The aim of this study was to develop and test a new Web-based rehabilitation tool that provides intensive cognitive training at home under clinical prescription and monitoring, at affordable costs.

**Methods:**

From a pool of 60 original exercises, designed and used over the past 10 years for cognitive training at our center, we developed 27 exercises on a computer game format, with automatic increase or decrease of difficulty levels. These exercises were assembled in a clean, user-friendly design and covered various cognitive domains such as attention (n=4), memory (n=11), language (n=3), calculus (n=3), praxis (n=2), and executive functions (n=3). A Web 2.0 platform was also designed to provide medical prescription of cognitive training sessions, performed at the patient’s home. These sessions included continuous monitoring of compliance, performance, and evolution; algorithms for automatic adjustment and long-term learning through use, and database recording of all activities. The end-user interaction test included 80 patients from our memory clinic from several groups including subjective memory complaints (n=20), traumatic brain injury (n=20), stroke and other static brain lesions (n=20), and mild Alzheimer’s disease (n=20). During a 1-hour session, patients and their relatives were taught to use the system and allowed to practice using it. At the end of the session, they were asked to complete a questionnaire.

**Results:**

A total of 48/80 patients (60%) attended the training session. The mean age of the patients was 60 years (SD 13.3, range 41-78), and the mean level of formal education was 6 years (range 4-16). Of all the participants, 32/48 patients (66%) have previously used a computer. All patients and their relatives made a positive evaluation of the cognitive training tool. Only 2/48 patients (4%) were not interested in performing the exercises at home; 19/48 patients (39%) mentioned the need for further coaching from a relative or health care professional. The patients who mentioned difficulties in performing the exercises have not used the computer earlier.

**Conclusions:**

This new Web-based system was very well accepted by patients and their relatives, who showed high levels of motivation to use it on a daily basis at home. The simplicity of its use and comfort were especially outlined. This tool will have an important effect on human resource management, in increasing the patient access to specialized health care and improving the quality and national health system costs of rehabilitation programs.

## Introduction

### Overview

Neurological disorders are commonly associated with a variety of cognitive and motor deficits that result in an ever increasing demand for health services. Among all major groups of diseases, neurological disorders constitute 6.3% of the global burden of diseases worldwide [1]. This value may be as high as 10.9% for high-income countries and 11.2% in the European region, which corresponds to 15-30 years of life lost adjusted for disability per 1000 inhabitants each year [1]. Irrespective of the cause (eg, stroke, brain injury, or dementia), people with cognitive impairments rarely recover spontaneously or completely [2].

Once established, brain damage is difficult to revert and pharmacological tools with a confirmed positive result are scarce [3-5]. The recovery process is typically slow, relies on the remaining plastic properties of brain tissue, and is highly dependent on complex and intensive assisted rehabilitation programs [6]. Similar to other rehabilitation processes, the results of the recovery process also depend on the timely onset, intensity, and specificity of the treatments [7,8].

The neurorehabilitation programs have proven efficacious in the compensation, improvement, and stabilization of cognitive deficits in several diseases and nosological models [9-17]. However, despite being accepted as a fundamental component of current treatment plans, these programs often impose strong restrictions on the patients’ access to such treatments [18,19]. These programs commonly require multidisciplinary teams and are usually performed in hospital settings, away from the patient’s home, in the presence of a relative (a huge effort by patients, families, and institutions). Classic cognitive training in particular requires pencil-and-paper tasks and object manipulation under specialized supervision. These characteristics result in a large economic burden to both the health system and families [20,21]. Furthermore, they limit the efficacy of the treatment by increasing the difficulty in coping with rehabilitation sessions in due time (soon after injury) and in attaining the high intensity of treatment necessary to foster nervous system plasticity [8,22].

The computer has emerged as a tool for the training and educating patients with brain injury, thus reducing the large demand for human resources and increasing the motivation of these patients [9,23]. However, the use of informatics programs in cognitive training is a recent approach; hence well-designed clinical trials are scarce, and the majority of them are inadequate for efficient integration in current clinical practice [18,24]. Therefore, the sole reliance on repeated exposure to computer-based tasks without some involvement and intervention by a therapist is not recommended [9].

To address these problems, we decided to develop a new integrative Web-based tool “COGWEB” for home-based cognitive training, centered not only on the patients’ needs but also on professionals and institutional requests. This instrument takes advantage of the growing knowledge on computerized training protocols for cognitive rehabilitation [18,25-29] and combines a Web-based platform with computer exercises developed over the last few years in an outpatient memory clinic. The aim of this tool is to increase the quality and overall intensity of cognitive training programs.

This paper presents the main characteristics of the developed system and the results of a usability testing of the online system performed by patients suffering from highly prevalent neurological diseases, and their relatives.

### Previous Approaches

Over the past few years, several solutions have been proposed to increase the availability of cognitive training. In fact, the market is flooded with commercial brain exercise programs that claim to improve cognition, have diagnostic abilities, and even replace the role of specialized health professionals along the way [30]. However, extensive clinical validation is still lacking and only a few programs have undergone scientific inquiry [26,27,31-33].

Previous approaches to cognitive training can be summarized into 3 categories: (1) cognitive domain-specific programs, (2) neuropsychological software programs, and (3) video games.

Cognitive domain-specific programs train specific cognitive capacities, usually under professional guidance at a health institution, and rely on the repetition of standardized tasks on a computer. Training of reaction time [34], processing speed [35], selective attention [36], and working memory [37,38] are some examples of these applications.

Neuropsychological software programs are designed to train several cognitive domains using a variety of tasks. These programs rely on immediate feedback and allow individuals to evolve based on their performance. Most studies with these tools analyze multiple cognitive domain interventions, either in the lab or remotely, for example, the Posit Science Brain Fitness Program [39], the Integrated Cognitive Stimulation and Training Program [40], the Neuropsychological Training [41], the CogniFit Personal Coach [42], and Lumosity [29]. Most of these programs are commercially available and especially designed for older people, as this age group is their biggest target market [30]. Nonetheless, there is a specific growing group dedicated to children and academic performance, usually under the format of tutorials [43].

Video games include computer or other electronic games where the players are enrolled in a set of activities oriented toward achieving a specific goal. Patients are given immediate feedback, and these games allow for an automatic progression between different levels of difficulty according to performance. The cognitive domains in this category are more difficult to individualize [18,44]. This category includes games originally designed to improve cognition like Nintendo’s Big Brain Academy for Wii [45], classics like Pac Man, Donkey Kong, or Tetris that were studied for processing speed [46], and recent commercial successes like Rise of Nations [47] and Medal of Honor [48] that were assessed for attention, memory, executive function, and visuospatial abilities.

### Unmet Needs and Problems

Computerized cognitive training offers several advantages over traditional pencil-and-paper tasks mediated by a psychologist or therapist at a health institution. The human resource costs per patient treated and the treatment time decrease, and home-bound or remotely located persons can have easier access to the treatment. In spite of all the available alternatives, several important needs remain unanswered [49]. Most of the programs available have a reductionist approach to brain conditioning or rehabilitation, discourage human relations in favor of self-executed exercises at home, and increase the distance between individuals or patients and specialized health professionals [30]. Furthermore, although all individuals may benefit from the use of novel technologies, the acquisition of regular training routines and computer skills is not straightforward for older people or patients with cognitive problems if we want to have an inclusive approach.

From the scientific point of view, research studies lack well-conducted randomized clinical trials, and most importantly, a clear definition of what a placebo is in these trials [50,51]. Furthermore, there is an absence of dose-finding studies that could assess what is necessary to obtain effects on other cognitive domains or improvement of daily living activities. Another major concern is the lack of studies to determine the possible side effects of these interventions. Noninvasive brain stimulation and cognitive enhancement strategies may have a mental cost on some abilities [52]. This feature is of utmost importance to deal with brain injury models like stroke or traumatic brain injury, where the rehabilitation of several brain functions competes for the uninjured cortex plastic and metabolic properties. Side effects are also important in neurodegenerative disease models where intensive training activities may have undesired effects if not controlled, monitored, or integrated in the social life and networks of individuals [30,44,49].

## Methods

### End-User Interaction Study Design

#### Clinical Settings for Using COGWEB

**Figure 1:**
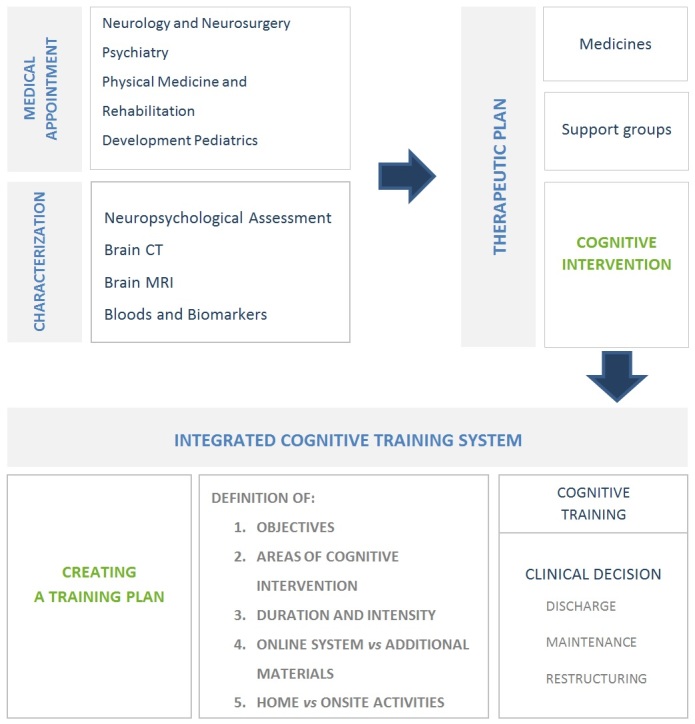
Clinical context in which COGWEB was developed.

Patients with changes in cognition attend different specialized medical appointments, where their medical and therapeutic needs are identified and assessed. Normally in the diagnosis procedure, besides other examinations, the patient is frequently submitted to neuropsychological assessments. Therapeutic plans for cognitive intervention are defined in this multidisciplinary clinical environment. The COGWEB appears in this context as a work tool, and the practitioner is responsible to manage and use the system, according to the clinical context and the patient’s characteristics (Figure 1, Table 1). The mandatory prerequisites for using the COGWEB are the existence of a medical diagnosis, a detailed neuropsychological characterization of the cognitive impairments, and a health professional willing to manage the treatment program, essential for a quality analysis of the system. The therapist that handles the system plays a central role in defining the degree of supervision and the type of patient exposure to the treatment and the Web-based system. The management of objectives, areas of cognitive intervention, individual composition of training sessions, and duration and intensity of treatment are all under the responsibility of the health professional. Although the presence of therapists is not continuously necessary for the training, they can actively direct all activities via online interaction and periodic (eg, daily, weekly, or monthly) meetings or telephone contact with the patient and caregiver (Multimedia Appendix 1).

**Table 1:** Target neurological conditions of the COGWEB system.

Major subgroups of diseases	Most important diseases
Non progressive structural lesions	Traumatic brain injury
	Stroke
	Sequels of brain paralysis, anoxia, radiotherapy, encephalitis, brain surgery, and other static brain lesions
Neurodegenerative diseases at an initial stage	Mild cognitive impairment
	Alzheimer’s disease
	Parkinson’s disease
	Vascular dementia
Cognitive dysfunction of functional nature	Subjective memory complaints
	Depression
	Normal aging and active aging strategies
Other nosological models	Multiple sclerosis
	Schizophrenia
	Hyperactivity and attention deficit (adults and children)

#### Patient Selection

A group of 80 consecutive patients from our outpatient memory clinic were selected and grouped equally into 4 nosological groups: 20/80 (25%) with subjective memory complaints, 25% with traumatic brain injury, 25% with stroke and other static brain lesions, and 25% with mild Alzheimer’s disease. The recruitment process took 3 months, and the following cumulative selection criteria were included: (1) a medical diagnosis compatible with 1 of the 4 groups, (2) 4 years of formal education completed, (3) favorable opinion of the attending neurologist, (4) no sensorial or physical deficiency preventing the use of regular computers unaided (eg, blindness, hemiplegia, or amputation), and (5) informed consent from both the patient and relative.

#### Group Sessions, Procedures, and Usability Questionnaire

Patients and caregivers (n≤20) were scheduled for psychoeducational group sessions at the hospital in a room with 10 computers with the Internet access. Two attempts per patient were performed to schedule the sessions at working hours. The sessions were structured into 3 parts (20 minutes each): (1) a psychologist provided an overview about the program and individual credentials for each patient to assess the online system; (2) the pair patient-caregiver was allowed to experiment with the program unaided on 1 of the 10 computers in the room; and (3) after completing a regular training session with 8 different exercises, an opinion questionnaire on the easiness of use and motivation for using it at home was answered anonymously by both the patient and caregiver in the absence of the psychologist (Table 2).

**Table 2:** Opinion questionnaire.

Question		Possible answers
Q1	Were the instructions easy to follow?	Yes/No
Q2	Were the exercises interesting to you?	Yes/No
Q3	Did you find the training useful to you?	Yes/No
Q4	Are you motivated to use it at home?	Yes/No
Q5	Having completed this training session, do you feel already independent to use it, or do you need additional training?	Independent/Additional training

#### Ethical Issues

All patients and caregivers understood the purpose of the study and provided written informed consent. An approval from the referring neurologist was also obtained to guarantee that patient and caregiver expectations were properly managed after the usability test. This study was approved by the hospital review board and ethics commission.

### Rehabilitation Tool

#### Main Characteristics

The Integrated Cognitive Training System is composed of 2 components: (1) an online platform, COGWEB and (2) a series of tools in the classic format of exercise books [53,54].

**Figure 2:**
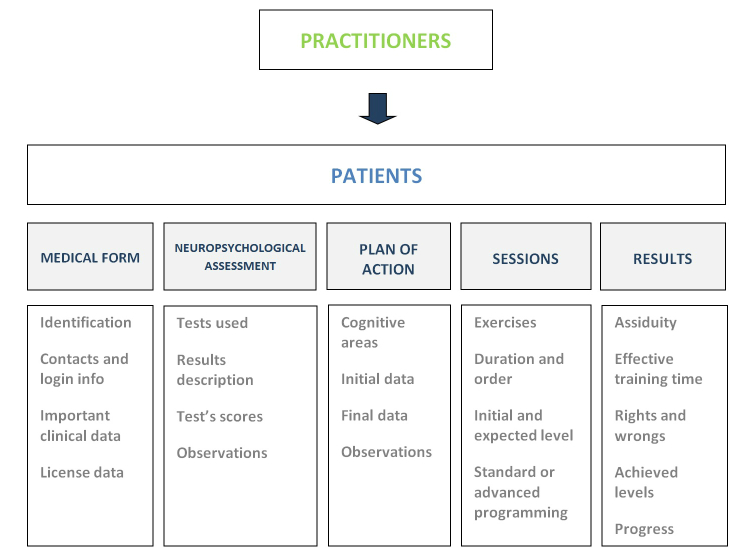
Global system scheme.

First, the COGWEB allows for the implementation of personalized cognitive training programs remotely, in the patient’s living environment. The tool is implemented through the professionally supervised prescription of exercise sessions, in computer game format, targeted to various cognitive functions, such as attention, executive functions, memory, language, praxis, gnosis, and calculus. Supervision is conducted by specialized practitioners without the loss of human contact or management (Figure 2).

Second, the exercise books were designed in parallel with the online platform and are useful during the initial stages of the training (Table 3). They can also be used to switch between stimulation methodologies if deemed necessary by the health professional, and to help people who, for various reasons, have no regular access to the Internet. Thus, people who face difficulty in using computers can start their training activities with paper and pencil, acquire routines, and then move up to a more intensive system (Multimedia Appendix 2).

Both system components are meant to be used as support for a wide range of cognitive interventional approaches, or at distance, under supervision with the neuropsychologist, being more or less present depending on the case (Figure 1, Table 1). The main structure of the online system is described in Figure 2 and its principal functional characteristics, resulted from a set of usability requirements defined by a board of clinicians, and are explained in the following text.

**Figure 3:**
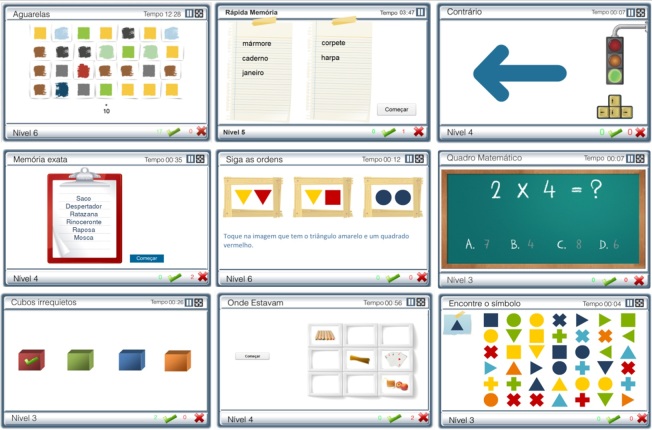
Examples of COGWEB exercises.

The online system was structured for modular cognitive training, as exercises were grouped according to major cognitive function stimulated (Table 4). This system covers different degrees of impairment, from normal function to moderate deficits, given that all exercises have sequential levels of difficulty and were designed for use in a wide range of diseases and ages (Figure 3). The monitoring tools coupled with biostatistics and long-term system analysis tools and a storage system that records performance continuously are incorporated into the system to supervise clinical evolution and adjust programs according to the patients’ progression. This system can be used in supervised group sessions with patients and can also be accessed at home or at any other place in the community with an Internet connection, only requiring a login information (without software installation or updates).

**Table 3:** Exercise books available and their target population.

Exercise books	Active ageing	Degenerative diseases			Static brain lesions	
		MCI^a^	Mild dementia	Moderate dementia	Stroke	TBI^b^
Weekly notebooks, Volumes I to IV	✓	✓	✓	✓	✓	✓
Monthly notebooks Level 3, Volumes I to III	✓	✓			✓	✓
Monthly notebooks Level 2, Volumes I to III			✓		✓	✓
Monthly notebooks Level 1, Volumes I to III				✓	✓	✓
COGWEB Art, 3D pieces	✓	✓	✓		✓	✓

^a^Mild cognitive impairment.

^b^Traumatic brain injury.

**Table 4:** Available exercises per cognitive domain.

Cognitive domain	Exercise	Levels (N)
Attention	Attention to the letter	5
	Attention to the number	5
	Find the letter	9
	Water colors	10
Memory	Attention to the news	3
	Fast eye	8
	Fast memory	8
	Long memory	14
	Numbers in order	8
	Restless cubes	7
	Reverse the stars	7
	Supermarket	8
	Who moved	7
	Worms	7
	Where were they	8
Language	Arrange the words	6
	Follow the orders	9
	Starts with	8
Executive functions	Match the color	3
	Contrary	7
	Inside or out	9
	Logic mind	9
Calculus	Fast mind	5
	Let’s go shopping	3
	Mathematical table	6
Constructive capacity	Puzzles	6
	COGWEB Art	7

#### Online System Architecture

The medical system, accessible via Internet browser, was designed to meet the requirements of health professionals and patients. Both groups need specialized and usable interfaces to access the system and introduce daily inputs. All data are centralized, recorded, and acceded on a health record system. During the cognitive training sessions performed by the patient, all of the data is updated continuously in real time via Web-based system and is saved on a remote database that furnishes all required information to be examined by the health professional. The database contains information of all patients and health professionals, and maintains a record of all training programs prescribed and patient performance. The system also includes a biostatistics analysis framework of unidentified data generated by the users of the system. The main purpose of the system is to analyze the quality of processes according to the standards established by a board of consulting clinicians. All data used for this computation remain unidentified and cannot be linked to the professional or patient who generated it. This policy was set according to the strict demands of the National Commission for Data Protection, established in accordance with European regulations (Directive 95/46/EC) [55]. The results from these computational operations are used to assess the application quality of the system by health professionals, to perform benchmarks of clinical outcomes, and to aid in the long-term improvement and context adaptation of the system. Some changes occur automatically, according to machine learning algorithms based on the internal statistical analysis. These tools provide additional evidence for the substitution of a useless exercise, the changing of system features that lead to errors, setting new automatisms like alert signs for some clinical situations, or the preparation of specific educational campaigns for professionals.

#### Network Operations and Coworking

The system is designed to become accessible to a large number of people, through a network of centers and practitioners specialized in using it. Upon agreement by the professionals and patients, anonymous data on the treatments prescribed and clinical evolution are stored on a centralized application. The analysis of these data allows the depiction of the trends and the assessment of the quality of the tools and operative processes throughout the time. Thus, the system can be evolved and adapted to the needs of patients, professionals, and institutions. All agents can share useful information and guidelines on operational processes through a collaborative network. Furthermore, all neuropsychologists and practitioners who receive training on using the COGWEB system and share quality criteria in operating the system are stimulated to develop personal and team research projects. This step will improve the quality of the projects, increase the size of possible samples without incurring additional costs, and conduct multicenter trials while shortening the required time to complete them.

#### Professional Console

##### Overview

The health professionals can access the rehabilitation program by entering their username and password on the website [56]. The website, in addition to allowing access to the online training area, contains educational content targeting the general population and a blog. The aim of the site is to provide scientific and pedagogical information about cognitive functioning and its changes, and the possibilities and indications for cognitive training [56].

The most important menu is the patient health record where the professional may add patient’s information to the system and manage it later. The patient menu includes several submenus: medical form, neuropsychological assessment, intervention plans, session programming, and results.

##### Medical Form

In this submenu, 3 types of data can be inserted: (1) identification data, (2) clinical data, and (3) data on the duration of the license to use the system and patients’ credentials to access it.

##### Neuropsychological Assessment

This menu, allowing the entry of neuropsychological assessment data on any patient, is organized into 2 parts. The first part records general descriptive data of the evaluation and the second part helps record each test and the quantitative results (preferably raw data) obtained in each neuropsychological test. Therefore, several evaluations can be recorded over time.

##### Intervention Plans

Health professionals can use this option to enter the general information of the treatment plan, like duration, main cognitive domains, and the expected intensity of training. This option allows to entering information on as many intervention plans as needed. The data entered in this section can be used for the detailed evaluation of the quality of the tasks prescribed to the patient in the next session. If a decision is made to train working memory or attention, the system provides information on all of the exercises the patient is prescribed, in addition to their duration.

##### Session Programming

The prescription system provides 2 ways to create sessions: (1) the standard mode and (2) the advanced mode. These two work modes share a set of prescription parameters, and the planning of each session requires the following parameters: the selection of exercises, their starting and completion dates, their order of appearance, the duration of exposure to each exercise, and the initial level and the level expected to be achieved at the end of the plan. These two modes, however, differ in defining and managing the training schedule. The standard mode is a faster and simpler way to plan sessions in which the patient conducts the same set of exercises in consecutive days. In the advanced prescription mode, the prescribing health professionals select the day on which they want a specific session to be active and the intended activation period, that is, morning, afternoon, or all day. Thus, the needs that currently exist in sophisticated and intensive rehabilitation or cognitive training methodologies in specific areas can be met.

##### Results

**Figure 4:**
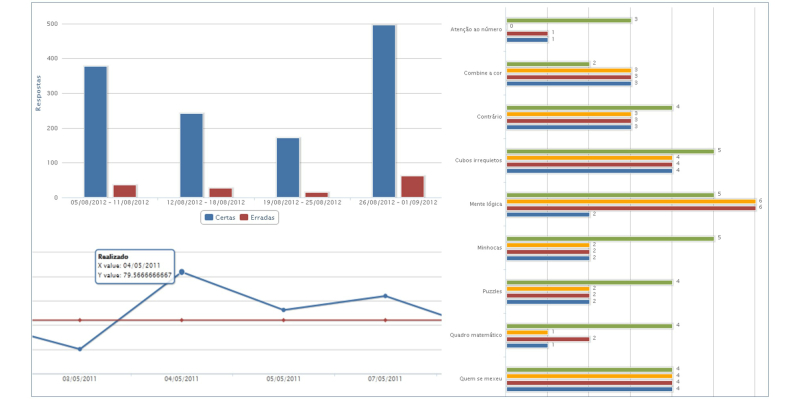
Examples of progress graphs.

The current system provides several kinds of progress graphs: right answers versus wrong answers, global training time (actual realized time vs planned time), game time in each exercise, concluded levels, accesses (realized accesses vs planned accesses), and a progress summary per exercise (expected performance vs real performance) (Figure 4).

#### Patient Console

##### Overview

The COGWEB is accessed through the website, similar to the professional’s login [56]. The entire login dynamic is identical in both the consoles with the only difference in the attributes and areas associated to the username/password.

##### Training Sessions

**Figure 5:**
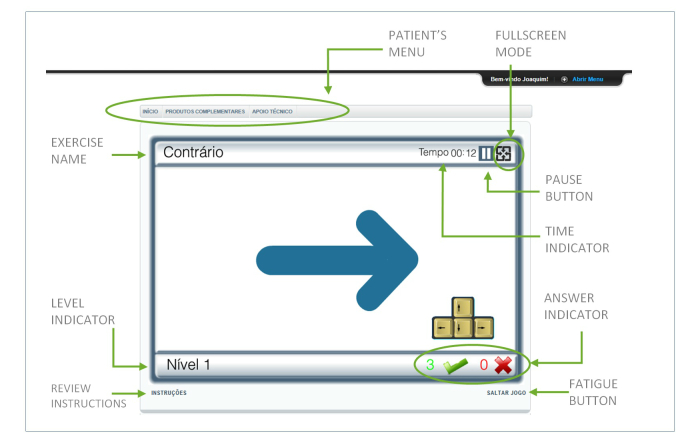
Screen appearance of the patient training area and principal features of the game arena.

As soon as the patient credentials are introduced, the training session starts automatically. First, the patient views a welcome screen with the training session information (number of exercises prescribed, total duration, and other general instructions) and a start button. When the patient feels ready, the first exercise planned starts, with no need for additional clicks or menu navigation. The simplicity of accessing the program eliminates the obstacles that may hinder training program compliance. The training arena is presented, by default, in a normal sized window. However, for added convenience during the exercise, the user can switch to full screen, by pressing the signed button (Figure 5).

##### Game Arena

Each exercise starts with a set of instructions, written on the screen and spoken through the speakers, for 20 seconds before the onset of the exercise. Instructions can be viewed at any time, even during the exercise, by pressing the instructions button. After the instructions, the exercise starts and the game arena appears. However, as time is an important factor in some of the exercises (eg, trainings processing speed), it is crucial to guarantee the patient’s attention to stimuli from the beginning. For that reason, there is a start button on the screen. The game arena is similar between various exercises making the learning and adaptation process easier. It comprises a central area, where the exercise takes place and the answers are given, with a frame around it. The exercise name is shown on the upper left hand corner of the frame, and the time remaining, the pause button, and the full screen activation button appear on the upper right hand corner. The lower left hand corner shows the level indicator and the bottom right the answer indicator (right and wrong) (Figure 5). The bottom of the screen displays 2 buttons: (1) the instructions button, which enables the patient to review the instructions even during the exercise and (2) the fatigue button (designated, skip game button), which can be activated if the patient feels tired or discouraged with a specific game.

The pause button allows the session to be stopped in case of unforeseen events that might distract the patient and hinder the performance. This button should be used exceptionally.

##### Motivation Tools

Upon successful completion of each level, a support message appears that is expressed simultaneously on the screen and in audio. When the performance by the end of the level does not fulfill the criteria for progression, the level is either maintained or decreased, depending on the progression rules for each game (see description of exercises). In this case, no information is given to the patient, the instructions for that level are just repeated, and the game continues. During the last exercise of each session, at the bottom of the game arena, the buttons that restart the session and end the session appear, which allow the patient to repeat the session, if they feel up to it, or end the session, in case they already feel fatigued. At the end of the session, this information is presented again.

#### Cognitive Exercises

##### Overview

The COGWEB system is composed of 27 independent exercises, distributed by different cognitive areas (Table 4). All exercises were first developed in a classic format including paper, pencil, cards, and other physical materials. Before being converted to the current computer game format, which took a period of more than 5 years, a pool of 60 original exercises were subject to extensive clinical use, validation, and refinement at our outpatient memory clinic [53,54]. These exercises share some important characteristics.

##### Functional Organization

Each exercise is organized around the stimulation of a specific cognitive area. However, one exercise does not train only one area, as other additional areas are also involved in solving the tasks. This multiplicity of tasks is intimately related to the integrated function characteristics of the human cognition.

##### Levels of Difficulty

The exercises were developed to train various degrees of cognitive defects, from mild to more severe impairments. Exercise progression is automatic, by levels, becoming more difficult or easier in response to the patient’s performance, both inside the same session or in consecutive sessions. The different degrees of difficulty, depending on each game, are obtained through manipulation of some of the features, either alone or in combination: the number of items per level, their complexity, and the interval between stimuli within the same level (game vs patient’s paced rhythms). For choosing the stimuli for each exercise (words, figures), special attention was given to various aspects that contribute to the complexity of the items, such as the extension of words, their degree of imageability, semantic proximity, or, in the case of figures, the number of graphic elements or graphic composition.

##### Random Stimuli

The structure of the exercises, at its base, is composed of sets of stimuli grouped by difficulty. On the same level of an exercise, the stimuli always appear in a random, nonsequential way to prevent memorization.

##### Information Sheets

**Figure 6:**
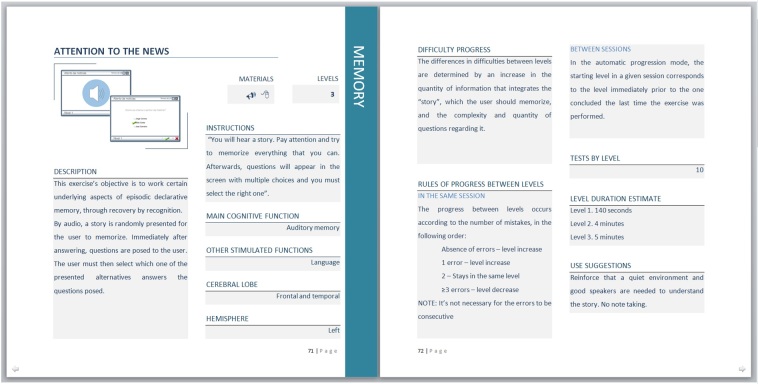
Example of an information sheet for the game exercise "Attention to the news".

For each exercise, the prescribing health professional has access to an individual sheet including the following parameters: general description, patient instructions, and multimedia requirements; main cognitive function that the game stimulates and other secondary stimulated functions; cortical areas recruited, according to anatomic-functional models of bibliographic basis; principles behind the choice of items that compose the game and the organization of their level of difficulty; the number of levels for each game, rules of progression between different levels and the number of tests in each level; estimated average time required for a normal individual to complete levels 1, 2, and 3 of each game (important for setting the minimum time of each game in sessions that might demand a level increase); and special use suggestions (Figure 6).

## Results

Of the 80 patients initially selected, 48 patients (60%) attended the psychoeducational group sessions and completed the usability test proposed. The mean age of the respondents was 60 years (SD 13.3, range 41-78). A total of 21/48 participants (44%) were female. The mean level of formal education was 6 years (SD 4.3, range 4-17). Previous use of the computer was shown by 32/48 patients (66%). Of all the participants, 32 patients (66%) did not complete the study because they were not able to attend the assessment sessions at the hospital. However, this did not differed between groups (*P*=.12).

**Figure 7:**
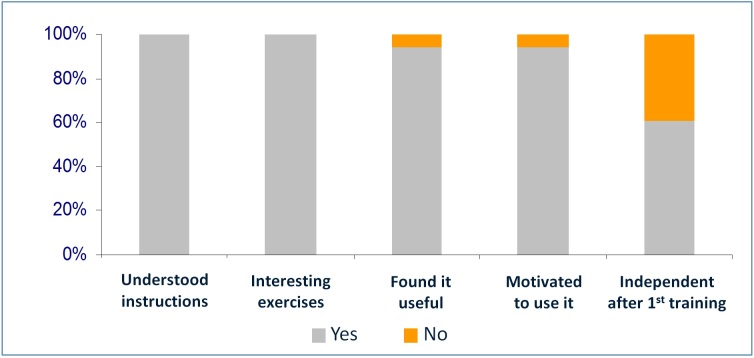
Answers to the opinion questionnaire (Q1-5) (%).

As shown in Figure 7, all patients in the presence of their caregivers understood the instructions given in the training session and during the execution of the 8 different exercises (Q1). All patients found the exercises used in the training sessions interesting to them (Q2). Only 2 of the 48 participants (4%) did not find the exercises useful for their clinical condition (Q3). The same percentage of respondents was not motivated to use the system on their own at home (Q4). After the first training session, 19/48 patients (39%) indicated the need for further help from the caregiver to use it at home (Q5).

In the group of patients that mentioned the need for additional training (n=19), 74% (14/19) were male, 79% (15/19) have not used the computer earlier, and 16% (3/19) had only sporadic exposure to computers.

## Discussion

### Principal Findings

This new system was very well received by patients and their relatives, who showed high levels of motivation to use it on a daily basis at home. The simplicity of its use and comfort were especially emphasized. Considering the mean age, the level of instruction, and the cognitive deficits of the patients enrolled, 19/48 patients (39%) required some kind of coaching to achieve independent use of the system. The formal evaluation of usability by both professionals and patients will be given further attention in future studies.

### Optimized Approaches

Compared to most related technologies available [18,43], COGWEB characteristics were defined after thorough study of existing cognitive training procedures in an outpatient memory clinic. The improvement of the quality and access to treatment by the patient were very important. Nonetheless, the system has the rehabilitation professional at its core as is recommended [9]. Ultimately, it is a specialized working tool that improves health decision making and time management per patient treated. A significant part of cognitive interventions can be done outside the health care units, by maintaining high quality levels of therapy through bidirectional communication between patients and professionals thus avoiding isolation. This step promotes patient comfort and treatment adhesion while eliminating economical and geographical barriers [30,44,49]. The impact of several degrees of personal presence of the therapist on the overall quality of training and outcomes are currently being evaluated in a prospective study.

Another significant feature of the system is its suitability for coworking and multicenter use in collaborative networks of professionals and institutions. This will foster investigation in the field and position COGWEB as one of the most prepared tools designed for clinical trials in cognitive interventional approaches. In the field of neurorehabilitation, high-quality scientific knowledge about several neurological and psychiatric diseases will be very important for treatment decision in the near future [5].

Finally, the incorporation of biostatistics and long-term system analysis tools are at the cornerstone of the system’s ability to improve quality and to adapt either to professionals, patients, institutional, or various clinical context needs or trends. This is similar to what can be achieved with some of the most advanced medication management information technology [57].

### Conclusions

We are now able to start clinical trials to test and measure intensive cognitive training protocols and to evaluate its positive and also possibly negative effects on a variety of diseases and settings [52,58]. The quantification of the economic impact and health gains of these strategies for health systems is also a priority [1]. The system will continue to evolve, basically with the development of new exercises and features to accommodate the needs from diverse populations and clinical settings of operation.

## Multimedia Appendix 1

Web-based cognitive training system presentation for patients.

## Multimedia Appendix 2

Exercise books presentation for patients.

## Abbreviations

COGWEB: Web-based cognitive training system

## References

[1] World Health Organization (2006). Neurological Disorders: Public Health Challenges.

[2] Katz DI, Polyak M, Coughlan D, Nichols M, Roche A (2009). Natural history of recovery from brain injury after prolonged disorders of consciousness: outcome of patients admitted to inpatient rehabilitation with 1-4 year follow-up. Prog Brain Res.

[3] Barbay S, Nudo RJ (2009). The effects of amphetamine on recovery of function in animal models of cerebral injury: a critical appraisal. NeuroRehabilitation.

[4] Rösser N, Flöel A (2008). Pharmacological enhancement of motor recovery in subacute and chronic stroke. NeuroRehabilitation.

[5] Cramer SC, Sur M, Dobkin BH, O'Brien C, Sanger TD, Trojanowski JQ, Rumsey JM, Hicks R, Cameron J, Chen D, Chen WG, Cohen LG, deCharms C, Duffy CJ, Eden GF, Fetz EE, Filart R, Freund M, Grant SJ, Haber S, Kalivas PW, Kolb B, Kramer AF, Lynch (2011). Harnessing neuroplasticity for clinical applications. Brain.

[6] Huda S, Rodriguez R, Lastra L, Warren M, Lacourse MG, Cohen MJ, Cramer SC (2008). Cortical activation during foot movements: II effect of movement rate and side. Neuroreport.

[7] Cramer SC (2008). Repairing the human brain after stroke: I. Mechanisms of spontaneous recovery. Ann Neurol.

[8] Van Peppen RP, Kwakkel G, Wood-Dauphinee S, Hendriks HJ, Van der Wees PJ, Dekker J (2004). The impact of physical therapy on functional outcomes after stroke: what's the evidence?. Clin Rehabil.

[9] Cicerone KD, Langenbahn DM, Braden C, Malec JF, Kalmar K, Fraas M, Felicetti T, Laatsch L, Harley JP, Bergquist T, Azulay J, Cantor J, Ashman T (2011). Evidence-based cognitive rehabilitation: updated review of the literature from 2003 through 2008. Arch Phys Med Rehabil.

[10] Cappa SF, Benke T, Clarke S, Rossi B, Stemmer B, van Heugten CM, Gilhus NE, Barnes MP, Brainin M (2010). Cognitive rehabilitation. European Handbook of Neurological Management. Volume 1.

[11] van Heugten C, Gregório GW, Wade D (2012). Evidence-based cognitive rehabilitation after acquired brain injury: a systematic review of content of treatment. Neuropsychol Rehabil.

[12] Olazarán J, Reisberg B, Clare L, Cruz I, Peña-Casanova J, Del Ser T, Woods B, Beck C, Auer S, Lai C, Spector A, Fazio S, Bond J, Kivipelto M, Brodaty H, Rojo JM, Collins H, Teri L, Mittelman M, Orrell M, Feldman HH, Muñiz R (2010). Nonpharmacological therapies in Alzheimer's disease: a systematic review of efficacy. Dement Geriatr Cogn Disord.

[13] Woods B, Aguirre E, Spector AE, Orrell M (2012). Cognitive stimulation to improve cognitive functioning in people with dementia. Cochrane Database Syst Rev.

[14] Rosti-Otajärvi EM, Hämäläinen PI (2011). Neuropsychological rehabilitation for multiple sclerosis. Cochrane Database Syst Rev.

[15] Kluwe-Schiavon B, Sanvicente-Vieira B, Kristensen CH, Grassi-Oliveira R (2013). Executive functions rehabilitation for schizophrenia: a critical systematic review. J Psychiatr Res.

[16] Gray SA, Chaban P, Martinussen R, Goldberg R, Gotlieb H, Kronitz R, Hockenberry M, Tannock R (2012). Effects of a computerized working memory training program on working memory, attention, and academics in adolescents with severe LD and comorbid ADHD: a randomized controlled trial. J Child Psychol Psychiatry.

[17] Ball K, Edwards JD, Ross LA, McGwin G (2010). Cognitive training decreases motor vehicle collision involvement of older drivers. J Am Geriatr Soc.

[18] Kueider AM, Parisi JM, Gross AL, Rebok GW (2012). Computerized cognitive training with older adults: a systematic review. PLoS One.

[19] Gross AL, Parisi JM, Spira AP, Kueider AM, Ko JY, Saczynski JS, Samus QM, Rebok GW (2012). Memory training interventions for older adults: a meta-analysis. Aging Ment Health.

[20] Carod-Artal FJ, Egido JA (2009). Quality of life after stroke: the importance of a good recovery. Cerebrovasc Dis.

[21] Vincent C, Desrosiers J, Landreville P, Demers L, BRAD Group (2009). Burden of caregivers of people with stroke: evolution and predictors. Cerebrovasc Dis.

[22] Rebok GW, Langbaum JB, Jones RN, Gross AL, Parisi JM, Spira AP, Kueider AM, Petras H, Brandt J (2012). Memory training in the ACTIVE study: how much is needed and who benefits?. J Aging Health.

[23] Fernández-Calvo B, Rodríguez-Pérez R, Contador I, Rubio-Santorum A, Ramos F (2011). Efficacy of cognitive training programs based on new software technologies in patients with Alzheimer-type dementia. Psicothema.

[24] Schoenberg MR, Ruwe WD, Dawson K, McDonald NB, Houston B, Forducey PG (2008). Comparison of functional outcomes and treatment cost between a computer-based cognitive rehabilitation teletherapy program and a face-to-face rehabilitation program. Prof Psychol.

[25] González-Abraldes I, Millán-Calenti JC, Balo-García A, Tubío J, Lorenzo T, Maseda A (2010). Accesibility and usability of computer-based cognitive stimulation: Telecognitio. Rev Esp Geriatr Gerontol.

[26] Tárraga L, Boada M, Modinos G, Espinosa A, Diego S, Morera A, Guitart M, Balcells J, López OL, Becker JT (2006). A randomised pilot study to assess the efficacy of an interactive, multimedia tool of cognitive stimulation in Alzheimer's disease. J Neurol Neurosurg Psychiatry.

[27] Franco MA, Bueno AY, Ortiz LA, Carrasco MM, Ballesteros JC (2002). Uso de las nuevas tecnologías como instrumentos de intervención en programas de psicoestimulación. Psiquiatría Geriátrica.

[28] Sternberg DA, Ballard K, Hardy JL, Katz B, Doraiswamy PM, Scanlon M (2013). The largest human cognitive performance dataset reveals insights into the effects of lifestyle factors and aging. Front Hum Neurosci.

[29] Zickefoose S, Hux K, Brown J, Wulf K (2013). Let the games begin: a preliminary study using attention process training-3 and lumosity brain games to remediate attention deficits following traumatic brain injury. Brain Inj.

[30] George DR, Whitehouse PJ (2011). Marketplace of memory: what the brain fitness technology industry says about us and how we can do better. Gerontologist.

[31] Ruff R, Mahaffey R, Engel J, Farrow C, Cox D, Karzmark P (1994). Efficacy study of thinkable in the attention and memory retraining of traumatically head-injured patients. Brain Inj.

[32] Shatil E, Metzer A, Horvitz O, Miller A (2010). Home-based personalized cognitive training in MS patients: a study of adherence and cognitive performance. NeuroRehabilitation.

[33] de Bruin ED, van Het Reve E, Murer K (2013). A randomized controlled pilot study assessing the feasibility of combined motor-cognitive training and its effect on gait characteristics in the elderly. Clin Rehabil.

[34] Bisson E, Contant B, Sveistrup H, Lajoie Y (2007). Functional balance and dual-task reaction times in older adults are improved by virtual reality and biofeedback training. Cyberpsychol Behav.

[35] Edwards JD, Wadley VG, Vance DE, Wood K, Roenker DL, Ball KK (2005). The impact of speed of processing training on cognitive and everyday performance. Aging Ment Health.

[36] Mozolic JL, Long AB, Morgan AR, Rawley-Payne M, Laurienti PJ (2011). A cognitive training intervention improves modality-specific attention in a randomized controlled trial of healthy older adults. Neurobiol Aging.

[37] Li SC, Schmiedek F, Huxhold O, Röcke C, Smith J, Lindenberger U (2008). Working memory plasticity in old age: practice gain, transfer, and maintenance. Psychol Aging.

[38] Brehmer Y, Westerberg H, Bäckman L (2012). Working-memory training in younger and older adults: training gains, transfer, and maintenance. Front Hum Neurosci.

[39] Smith GE, Housen P, Yaffe K, Ruff R, Kennison RF, Mahncke HW, Zelinski EM (2009). A cognitive training program based on principles of brain plasticity: results from the Improvement in Memory with Plasticity-based Adaptive Cognitive Training (IMPACT) study. J Am Geriatr Soc.

[40] Eckroth-Bucher M, Siberski J (2009). Preserving cognition through an integrated cognitive stimulation and training program. Am J Alzheimers Dis Other Demen.

[41] Cavallini E, Dunlosky J, Bottiroli S, Hertzog C, Vecchi T (2010). Promoting transfer in memory training for older adults. Aging Clin Exp Res.

[42] Peretz C, Korczyn AD, Shatil E, Aharonson V, Birnboim S, Giladi N (2011). Computer-based, personalized cognitive training versus classical computer games: a randomized double-blind prospective trial of cognitive stimulation. Neuroepidemiology.

[43] Rabipour S, Raz A (2012). Training the brain: fact and fad in cognitive and behavioral remediation. Brain Cogn.

[44] Bavelier D, Davidson RJ (2013). Brain training: Games to do you good. Nature.

[45] Ackerman PL, Kanfer R, Calderwood C (2010). Use it or lose it? Wii brain exercise practice and reading for domain knowledge. Psychol Aging.

[46] Clark JE, Lanphear AK, Riddick CC (1987). The effects of videogame playing on the response selection processing of elderly adults. J Gerontol.

[47] Basak C, Boot WR, Voss MW, Kramer AF (2008). Can training in a real-time strategy video game attenuate cognitive decline in older adults?. Psychol Aging.

[48] Green CS, Bavelier D (2003). Action video game modifies visual selective attention. Nature.

[49] Bavelier D, Green CS, Han DH, Renshaw PF, Merzenich MM, Gentile DA (2011). Brains on video games. Nat Rev Neurosci.

[50] Klingberg T (2010). Training and plasticity of working memory. Trends Cogn Sci.

[51] Owen AM, Hampshire A, Grahn JA, Stenton R, Dajani S, Burns AS, Howard RJ, Ballard CG (2010). Putting brain training to the test. Nature.

[52] Iuculano T, Cohen Kadosh R (2013). The mental cost of cognitive enhancement. J Neurosci.

[53] Cruz VT, Pais J (2012). Cogweb® - Sistema Integrado De estimulação Cognitiva: Manual de Formação Para Profissionais.

[54] Cruz VT, Pais J (2013). Cogweb® - Sistema Integrado de Estimulação Cognitiva: Manual de Bolso.

[55] European Comunnity (1995). Directive 95/46/EC of the European Parliamentof the Council of 24 October 1995 on the protection of individuals with regard to the processing of personal dataon the free movement of such data. Official Journal of the EC.

[56] Cruz VT, Pais J, Bento VF, Mateus C, Colunas M, Alves I (2013). Neuroinova, Lda.

[57] McKibbon KA, Lokker C, Handler SM, Dolovich LR, Holbrook AM, O'Reilly D, Tamblyn R, Hemens BJ, Basu R, Troyan S, Roshanov PS (2012). The effectiveness of integrated health information technologies across the phases of medication management: a systematic review of randomized controlled trials. J Am Med Inform Assoc.

[58] Langenbahn DM, Ashman T, Cantor J, Trott C (2013). An evidence-based review of cognitive rehabilitation in medical conditions affecting cognitive function. Arch Phys Med Rehabil.

